# Wnt Signaling Cross-Talks with JH Signaling by Suppressing *Met* and *gce* Expression

**DOI:** 10.1371/journal.pone.0026772

**Published:** 2011-11-08

**Authors:** Mohamed Abdou, Cheng Peng, Jianhua Huang, Ola Zyaan, Sheng Wang, Sheng Li, Jian Wang

**Affiliations:** 1 Department of Entomology, University of Maryland at College Park, College Park, Maryland, United States of America; 2 Key Laboratory of Insect Developmental and Evolutionary Biology, Institute of Plant Physiology and Ecology, Shanghai Institutes for Biological Sciences, Chinese Academy of Sciences, Shanghai, China; New Mexico State University, United States of America

## Abstract

Juvenile hormone (JH) plays key roles in controlling insect growth and metamorphosis. However, relatively little is known about the JH signaling pathways. Until recent years, increasing evidence has suggested that JH modulates the action of 20-hydroxyecdysone (20E) by regulating expression of *broad* (*br*), a 20E early response gene, through Met/Gce and Kr-h1. To identify other genes involved in JH signaling, we designed a novel *Drosophila* genetic screen to isolate mutations that derepress JH-mediated *br* suppression at early larval stages. We found that mutations in three Wnt signaling negative regulators in *Drosophila*, *Axin* (*Axn*), *supernumerary limbs* (*slmb*), and *naked cuticle* (*nkd*), caused precocious *br* expression, which could not be blocked by exogenous JHA. A similar phenotype was observed when *armadillo* (*arm*), the mediator of Wnt signaling, was overexpressed. qRT-PCR revealed that *Met*, *gce* and *Kr-h1*expression was suppressed in the *Axn*, *slmb* and *nkd* mutants as well as in *arm* gain-of-function larvae. Furthermore, ectopic expression of *gce* restored *Kr-h1* expression but not *Met* expression in the *arm* gain-of-function larvae. Taken together, we conclude that Wnt signaling cross-talks with JH signaling by suppressing transcription of *Met* and *gce*, genes that encode for putative JH receptors. The reduced JH activity further induces down-regulation of *Kr-h1*expression and eventually derepresses *br* expression in the *Drosophila* early larval stages.

## Introduction

Juvenile hormone (JH) is a critical hormone that regulates many aspects of insect physiology. One main role of JH is its classic “status quo” action in the regulation of insect development. When 20-hydroxyecdysone (20E) induces molting during early developmental stages, the presence of JH ensures that the molt results in a repeat of the previous stage [1], [2]. Therefore, JH does not block the 20E-coordinated molting process, but rather directs the action of 20E. During the last two decades, studies on the hormonal regulation of insect development have focused on understanding the molecular basis of 20E, JH, and their interaction.

At the molecular level, 20E binds to its heterodimer receptor, EcR/USP, to directly activate the transcription of a small set of early-response genes that encode transcriptional factors. These genes transduce and amplify the original hormonal signal by activating a large number of late-response genes that encode tissue-specific effector proteins necessary for insect molts and metamorphosis [3]. One of the 20E-induced early genes, *broad* (*br*), was identified as a key regulator in mediating the cross-talk between the 20E and JH signaling pathways. *Drosophila br* encodes four transcriptional factors that contain a common N-terminal domain and four pairs of different C2H2 DNA-binding zinc finger domains [4], [5]. The Br proteins directly regulate the transcription of 20E-induced late genes and are essential for the specification of pupal development [6], [7]. Consistent with its function, the Br proteins are predominantly expressed during the larval-pupal transition in all of the examined holometabolous insects [8]. Previous studies in *Manduca*, *Bombyx*, and *Tribolium* suggested that the temporal pattern of *br* expression results from the 20E and JH interaction. 20E directly induces *br* expression, which can be prevented by JH in young larvae [9]–[11]. Here, we demonstrate that JH is also required to repress *br* expression during early larval stages in *Drosophila*.

JH transduces its signal through Methoprene-tolerant (Met), Germ cell-expressed (Gce) and Krüppel-homolog 1 (Kr-h1) and the p160/SRC/NCoA-like molecule (Taiman in *Drosophila* and FISC in *Aedes*). The *Drosophila Met* and *gce* genes encode two functionally redundant bHLH-PAS protein family members, which have been proposed to be components of the elusive JH receptor [12]–[14]. Both *Met* and *gce* mutants are viable and resistant to JH analogs (JHA) as well as to natural JH III [14], [15]. However, *Met*-*gce* double mutants are prepupal lethal and phenocopies CA-ablation flies [14], [16], [17]. The Met protein binds JH III with high affinity [18], [19]. In *Tribolium*, suppression of *Met* activity by injecting double-stranded (ds) *Met* RNA causes precocious metamorphosis [20]. Kr-h1 is considered as a JH signaling component working downstream of Met. In both *Drosophila* and *Tribolium*, *Kruppel-homolog1* (*Kr-h1*) mRNA exhibits high levels during the embryonic stage and is continuously expressed in the larvae; then, it disappears during pupal and adult development [21]–[23]. *Kr-h1* expression can be induced in the abdominal integument by exogenous JH analog (JHA) at pupariation [22]. Suppression of *Kr-h1* by dsRNA in the early larval instars of *Tribolium* causes precocious *br* expression and premature metamorphosis after one succeeding instar [23]. Thus, Kr-h1 is necessary for JH to maintain the larval state during a molt by suppressing *br* expression. Studies in *Aedes*, *Drosophila* and *Tribolium* have demonstrated that the p160/SRC/NCoA-like molecule is also required for JH to induce expression of *Kr-h1* and other JH response genes [24], [25]. For example, *Aedes* FISC forms a functional complex with Met on the JH response element in the presence of JH and directly activates transcription of JH target genes [24].

In an attempt to isolate other genes involving JH signaling, we conducted a novel genetic screen and identified that mutations in three Wnt signaling component genes, *Axin* (*Axn*), *supernumerary limbs* (*slmb*), and *naked cuticle* (*nkd*), induced precocious *br* expression, which was similar to a loss of JH activity. The evolutionarily conserved Wnt signaling pathway controls numerous developmental processes [26]. The key mediator of the *Drosophila* Wnt pathway is Armadillo (Arm, the homolog of vertebrate β-catenin). When the Wnt signaling ligand, Wingless (Wg), is absent, the destruction complex is active and phosphorylates Arm, earmarking it for degradation. Upon Wg stimulation, the destruction complex is inactivated; as a result, unphosphorylated Arm accumulates in the cytosol and is targeted to the nucleus to stimulate transcription of Wnt target genes [27]. Many players in the Wnt signaling pathway negatively regulate its activity. For example, Axin (Axn) is one of the main components of the destruction complex [28]. Supernumerary limbs (Slmb) recognizes phosphorylated Arm and targets it for polyubiqitination and proteasomal destruction [29]. Naked cuticle (Nkd) antagonizes Wnt signaling by inhibiting nuclear import of Arm [30]. Our investigations reveal that the high activity of Wnt signaling in the *Axn*, *slmb*, and *nkd* mutants suppresses the transcription of *Met* and *gce*, genes encoding for putative JH receptors, thus linking Wnt signaling to JH signaling and insect metamorphosis for the first time.

## Results

### *GAL4-PG12* recapitulates the *br* expression pattern

**Figure 1:**
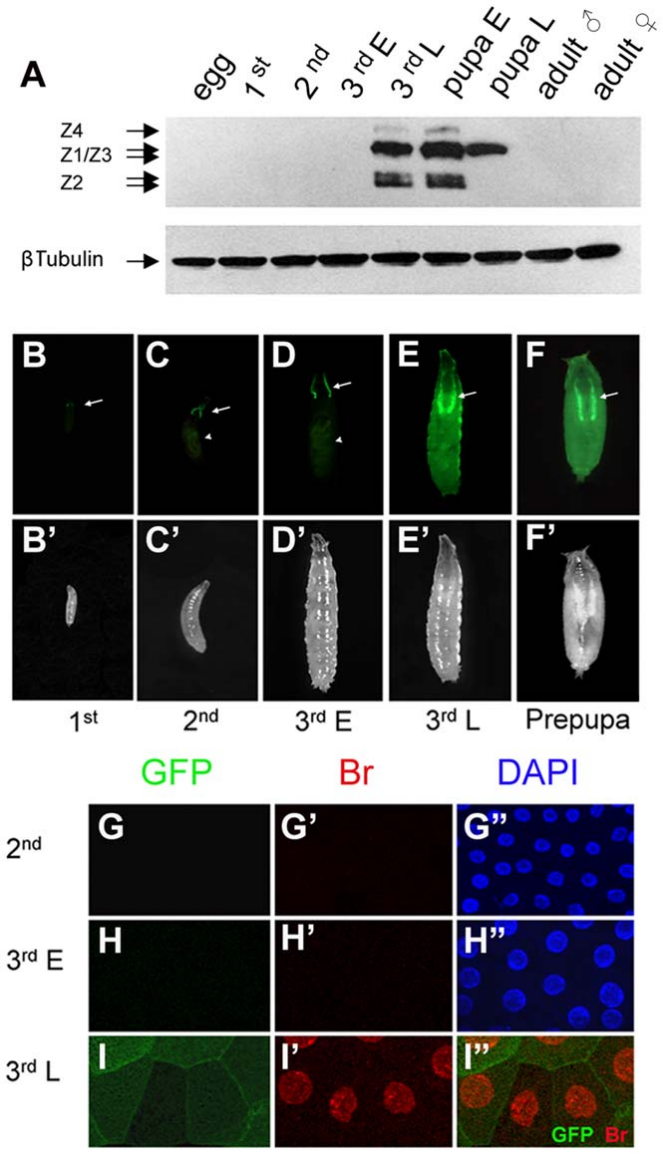
*GAL4-PG12* resembles endogenous *br* expression patterns. (A) Protein extracts isolated from wild type animals at different developmental stages were separated by SDS-PAGE. Br proteins were assessed by Western blotting using a Br-core antibody. Tubulin-β was used as a loading control. The Br proteins were only detected in the late 3^rd^ instar larval stage to pupal stage. All Br isoforms were expressed in the late 3^rd^ instar larvae and early pupae, but only Z1 and/or Z3 isoforms were expressed in the late pupae. (B–F) Expression of *GAL4-PG12* was marked by *GAL4-PG12,UAS-mCD8GFP*. Constitutive expression of *GAL4-PG12* in salivary glands (arrows) and auto-fluorescence of fly food in the midgut (arrowheads) are indicated. In tissues other than those from the salivary gland, *GAL4-PG12/UAS-mCD8GFP* was only expressed in late 3^rd^ instar larval and during early pupal stages (G and H). (B′–F′) White light images of the same organisms are shown in [B–F]. (G–I) *GAL4-PG12* expression was monitored by mCD8GFP (green) [G–I]. Endogenous Br proteins were recognized by a Br-core antibody (red) [G′–I′] and nuclei were marked with DAPI (blue) [G″–H″]. Neither endogenous Br nor *GAL4-PG12* were expressed in FB of the 2^nd^ instar or early 3^rd^ instar [G-G″ and H-H″], but both were expressed in FB of the late 3^rd^ instar [I-I″]. [I″] is a merged image of [I] and [I′].

It is well documented that *br* is a molecular marker for pupal commitment and specifies the larval-pupal metamorphosis in a variety of holometabolous insect species [31]. Western blotting using a *Drosophila* Br-core antibody, which recognizes all 4 Br isoforms [32], showed that Br proteins were highly expressed in late 3^rd^ instar larvae and pupae. Conversely, no Br proteins were detected from the embryonic stages to early 3^rd^ instar larval stag^es^ or in adults. Interestingly, during the larval-pupal metamorphosis, different Br isoforms exhibited distinct expression profiles, with all 4 isoforms (Z1, Z2, Z3, and Z4) expressed from the late 3^rd^ instar to early pupal stages and only 1 or 2 isoforms (Z1 and/or Z3) expressed in the late pupal stage (Fig. 1A).

**Figure 2:**
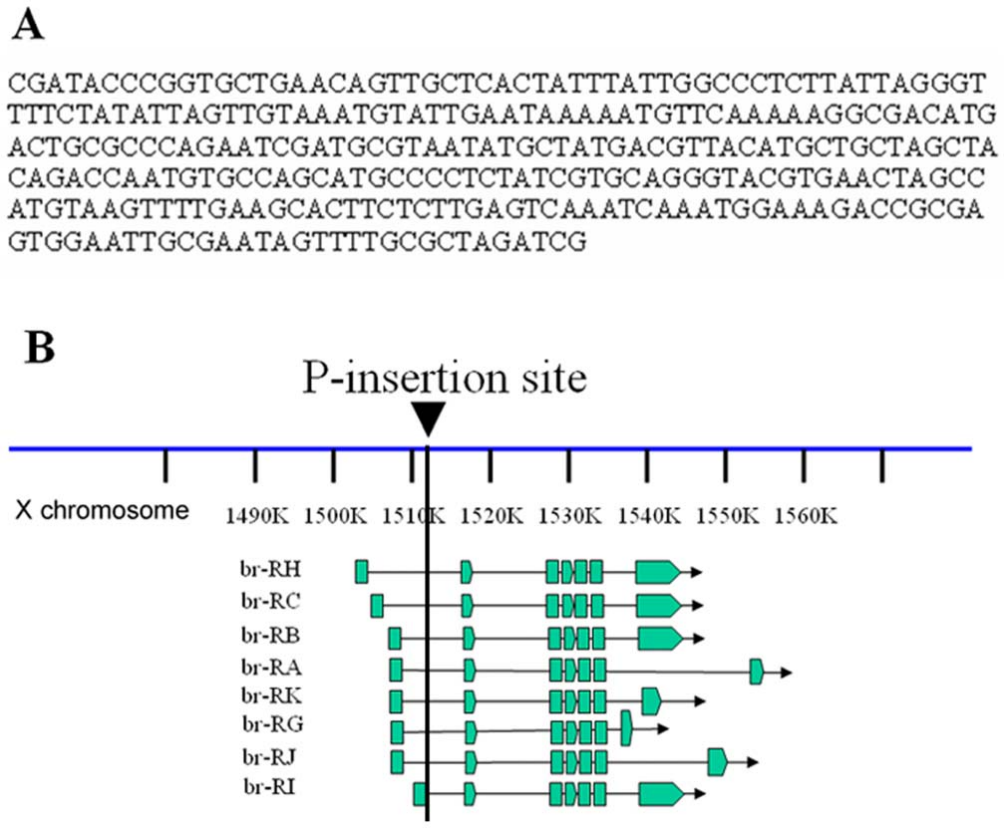
*GAL4-PG12* carries a *P*-element insertion in the first intron of *br* gene. (A) The flanking sequence of the *GAL4-PG12 P*-element insertion site identified by inverse PCR analysis. (B) The insertion site of *GAL4-PG12* was located within the first intron of the *br* gene by comparing the sequence with the *Drosophila* genome.

To monitor *br* expression in live organisms, we examined the expression patterns of GAL4 enhancer-trap lines inserted near the *br* gene. One of these lines, *GAL4-PG12*, closely resembled the temporal and spatial expression pattern of the endogenous *br* gene in tissues other than the salivary gland. In 1^st^, 2^nd^, and early 3^rd^ instar larval stages of *GAL4-PG12,UAS-mCD8GFP*, GFP expression was only detected in the salivary gland (Fig. 1B–D). This expression of *GAL4-PG12* in the salivary gland is a common feature for most GAL4 lines derived from the *P*{*GawB*} construct, which may carry a position-dependent, unidentified salivary gland enhancer [33]. However, in late 3^rd^ instar larvae and early pupae, an intensive GFP signal was observed in the whole organism (Fig. 1E and F). Inverse PCR analysis revealed that *GAL4-PG12* carries a *P*{*GawB*} construct within the first intron of the *br* gene (Fig. 2).

We next compared the expression pattern of *GAL4-PG12,UAS-mCD8GFP* with that of the *br* gene in the larval fat body (FB). Neither endogenous Br proteins nor GFP were detectable in the FB of 2^nd^ and early 3^rd^ instar larvae (Fig. 1G and H). In late 3^rd^ instar larvae, the Br proteins (red) were observed in the FB nuclei in the same cells as mCD8GFP (green), the cell membrane-attached marker driven by *GAL4-PG12* (Fig. 1I-I″). These results indicate that *GAL4-PG12* can be used to monitor endogenous *br* expression.

### JH represses *br* expression at early larval stages

To determine whether JH represses *br* expression in early *Drosophila* larval instars, we generated a transgenic fly line that harbors *juvenile hormone esterase* (*jhe*) cDNA driven by a heat-shock promoter (*hs-jhe*). JH is a common name for a family of sesquiterpenoid esters of methanol and hydrolysis of the conjugated methyl ester is generally regarded as one of the key pathways for inactivating the hormone [34]. JHE was reported to be the only esterase that hydrolyzes all types of JH in *Drosophila* [35]. Therefore, we expected that overexpression of *jhe* during early larval stages would reduce the JH titer in the hemolymph.

**Figure 3:**
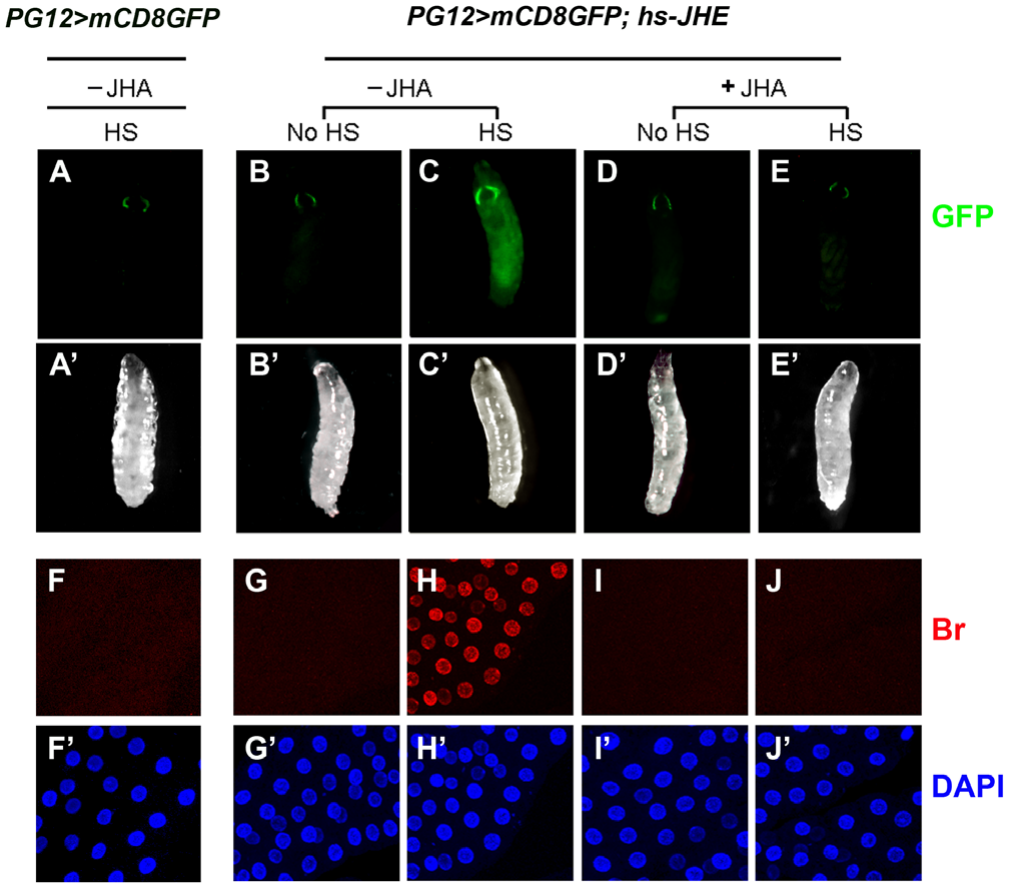
Ectopic expression of JHE induces precocious *br* expression in the 2^nd^ instar larvae. Flies carrying two copies of *hs-jhe* transgenes (*GAL4-PG12, UAS-mCD8GFP/Fm7C; hs-jhe^1^, hs-jhe^2^/+*) were reared on normal (−JHA) or 0.1 ppm pyriproxifen-containing (+JHA) food and were treated with (HS) or without (no-HS) heat shocking twice a day for 40 min at 37°C. *Br* expression was monitored by *GAL4-PG12,UAS-mCD8GFP* [A–D] and FB Br-core antibody staining in 2^nd^ instar larvae [E–H]. Precocious *br* expression occurred in 2^nd^ instar larvae that were reared on normal food and treated with heat shocking [B-B′ and F-F′]. However, this phenotype was blocked by JHA treatment [D-D′ and H-H′].

As the control, heat shock did not induce *br* expression in the *GAL4-PG12,UAS-mCD8GFP* 2^nd^ instar larvae (Fig. 3A and F). However, when *GAL4-PG12,UAS-mCD8GFP; UAS-JHE* 2^nd^ instar larvae were treated by heat shock, , precocious *br* expression was observed: levels of endogenous Br proteins increased (Fig. 3H), as did expression of the *GAL4-PG12,UAS-mCD8GFP* reporter (Fig. 3C). Nevertheless, when *hs-jhe* larvae were reared on food containing 0.1 ppm pyriproxifen, an efficient JH agonist (JHA) that is chemically different from natural JH [36], precocious *br* expression in the *hs-jhe* larvae was undetectable (Fig. 3E and J). Together, these results demonstrate that JH is required to suppress *br* expression during early larval stages in *Drosophila*.

### A genetic screen for mutations affecting *br* expression

**Figure 4:**
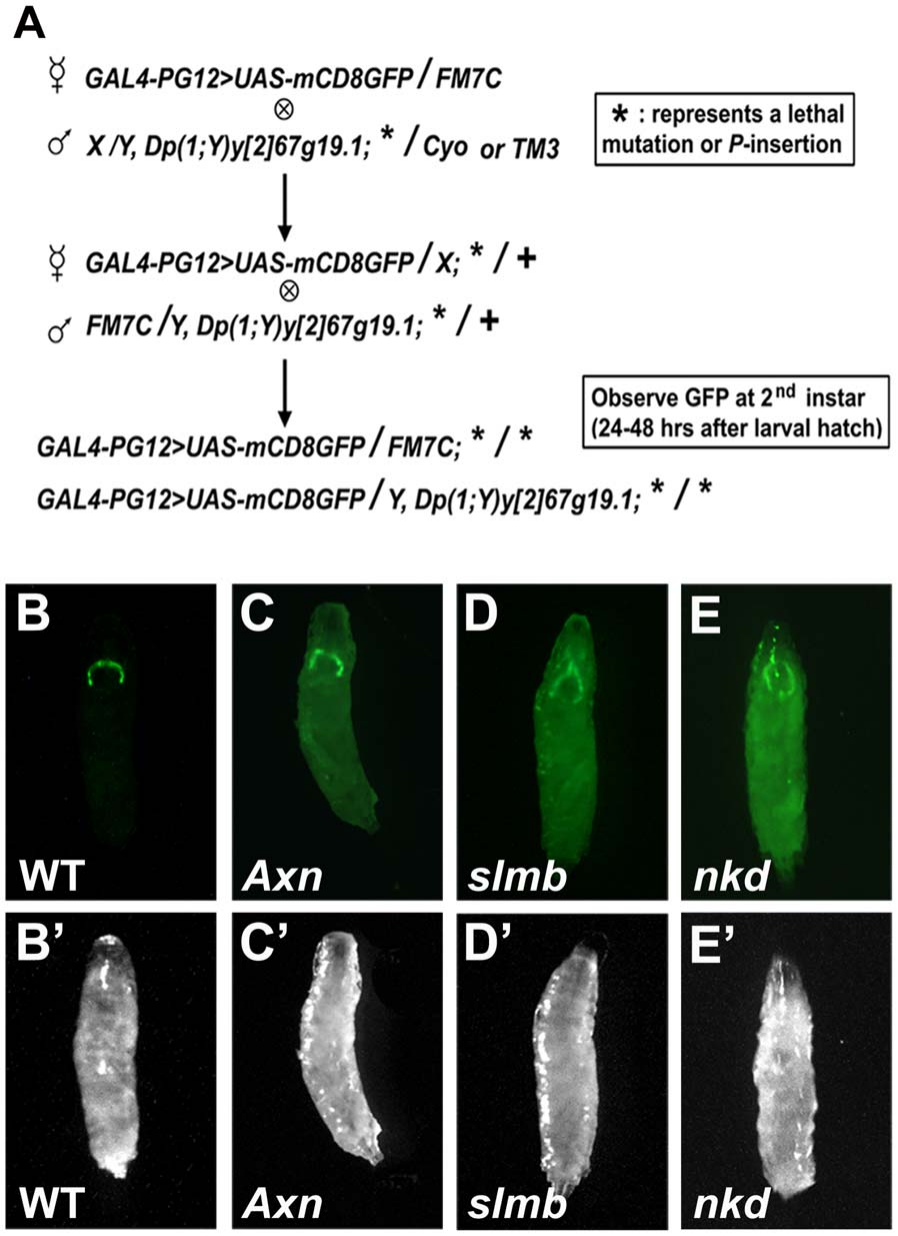
A genetic screen identifies that Axn, Slmb and Nkd regulate *br* expression. (A). Schematic diagram of genetic crosses for isolating mutations that derepress *br* expression in young larvae. *GAL4-PG12,UAS-mCD8GFP* (X chromosome) was used to monitor *br* expression. The lethal mutation or *P*-insertion on the 2^nd^ or 3^rd^ chromosome is represented by an asterisk (*). (B–E). GFP images show the expression of *GAL4-PG12,UASmCD8GFP* in 2^nd^ instar larvae. GFP was only expressed in the salivary gland of the wild type [B], but widely expressed in all tissues of *Axn^EY10228^* [C], *slmb^00295^* [D], and *nkd^2^* [E] mutant larvae. (B′–E′) White light images of the same organisms are shown in [B–E].

Because JH represses *br* expression during early larval stages, we reasoned that mutations that reduce the JH titer or disrupt JH action should cause precocious *br* expression in *Drosophila*. Accordingly, we designed and conducted a genetic screen to isolate genes that affect these processes. In these screens, *GAL4-PG12*,*UAS-mCD8GFP* on the X chromosome was used as a reporter of *br* expression, and lethal mutations or *P*-insertions on the 2^nd^ or 3^rd^ chromosome were made homozygous and screened for precocious *br* expression (Fig. 4A). Because most of the lethal lines allowed organisms to develop to early larval stages, we were able to examine GFP expression in the 2^nd^ instar under the fluorescent microscope. From 4,400 lethal lines, 55 mutations were isolated based on GFP expression in the 2^nd^ instar larvae. Genes associated with these mutations encode proteins with various molecular functions, including enzymes, signal transduction molecules, and transcriptional factors.

This genetic screen was efficient in identifying the genes required for JH biosynthesis. It not only isolated genes that are known to be involved in JH biosynthesis, such as *farnesyl diphosphate synthase* (*Fpps*) [37], *apterous* (*ap*) [38], *Insulin receptor* (*InR*) [39], [40], and N-*methyl*-d-*aspartate receptor 1* (*Nmdar1*) [41], but also revealed that Dpp-mediated TGF-β signaling in the corpus allatum stimulates JH biosynthesis by upregulating transcription of *JH acid methyltransferase* (*jhamt*), a key regulatory enzyme of JH synthesis [42]. The same genetic screen also isolated genes that are involved in JH signaling, such as *Kr-h1*. Another known JH signaling component, Met, was not identified by this screen because the *Met* gene is located to X chromosome. A reverse genetic study showed that precocious *br* expression was also detectable in *Met* mutant larvae [42].

### Mutations in the Wnt signaling negative regulators cause precocious *br* expression

Three important components of Wnt signaling, *Axn*, *slmb*, and *nkd* were found among these 55 genes. As shown in Fig. 4, expression of *GAL4-PG12*,*UAS-mCD8GFP* was restricted to salivary glands in the wild type 2^nd^ instar larvae (Fig. 4B), but ubiquitous expression of *GAL4-PG12*,*UAS-mCD8GFP* was detected at the same stage in the *Axn*, *slmb*, and *nkd* mutant larvae (Fig. 4C–E). These results suggest that Wnt signaling is required to repress *br* expression during the early larval stages, possibly by regulating either the JH titer or JH signaling.

### Exogenous JHA does not prevent precocious *br* expression in *Axn*, *slmb*, and *nkd* mutants

**Figure 5:**
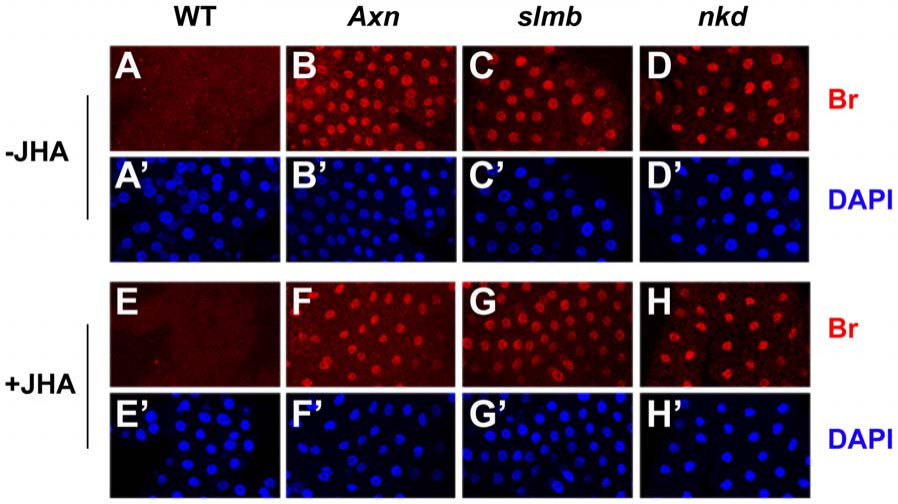
Precocious *br* expression in *Axn*, *slmb* and *nkd* mutants is not prevented by JHA. *Oregon* R, *Axn^EY10228^*, *slmb^00295^*, and *nkd^2^* mutants were reared on normal (−JHA) or 0.1 ppm pyriproxifen-containing (+JHA) food. Fat bodies of the 2^nd^ instar larvae were stained with a Br-core antibody (red). Nuclei were labeled with DAPI (blue).

Consistently, precocious *br* expression was observed when we used Br-core antibody staining at the 2^nd^ instar. Endogenous Br proteins were not detectable in the fat body (FB) of the wild type (Fig. 5A), but were observed in the FB nuclei of the *Axn^EY10228^*, *slmb^00295^*, and *nkd^2^* larvae (Fig. 5B–D). We then examined other *Axn*, *slmb*, and *nkd* alleles, including *Axn^16–21^*, *slmb^EY09052^*, and *nkd^3^*. Precocious *br* expression was detected in all cases.

Next, we asked whether the precocious *br* expression phenotype of the *Axn*, *slmb*, and *nkd* mutants could be blocked by exogenous JHA. Wild type, *Axn^EY10228^*, *slmb^00295^*, and *nkd^2^* larvae were reared on a diet containing 0.1 ppm pyriproxifen. Immunohistochemical results revealed that precocious *br* expression was not suppressed by exogenous JHA in the FB of the *Axn*, *slmb*, and *nkd* mutant larvae (Fig. 5F–H).

These results are opposite to what we observed in mutants that affect JH biosynthesis, such as *tkv* and *mad*, in which the precocious *br* expression was totally suppressed by exogenous JHA [42]. In contrast, these data are consistent with what we observed in the mutations that affect JH signaling, such as *Kr-h1* and *Met* [14], [42]. Therefore, we suggest that *Axn*, *slmb*, and *nkd* affect *br* expression by affecting JH signaling.

### Precocious *br* expression occurs in *Axn* mutants in a tissue-specific manner

**Figure 6:**
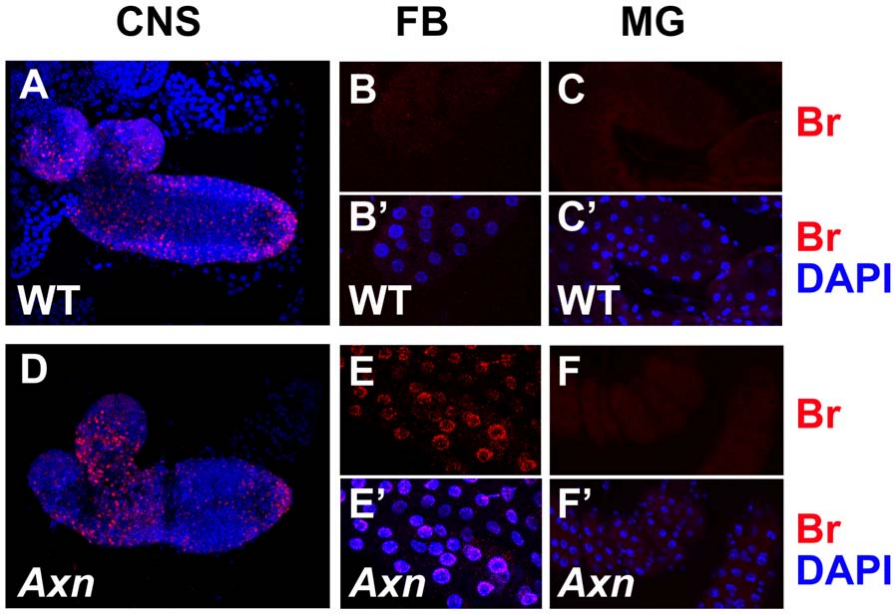
Precocious *br* expression occurs in *Axn* mutants in a tissue-specific manner. 2^nd^ instar larvae of *Oregon* R and *Axn^EY10228^* were dissected and stained with a Br-core antibody (red). Nuclei were labeled with DAPI (blue). Images show central nervous system (CNS) (Fig. 6A and D), fat body (FB) (Fig. 6B and E) and midgut (MG) (Fig. 6C and F).

We further detected *br* expression in different tissues of the wild type and *Axn* mutant 2^nd^ instar larvae. We found that *br* expressed in some of cells within the brain of wild type 2^nd^ instar larvae (Fig. 6A). The number and pattern of the *br* expressed-cells in the brain of *Axn* mutant larvae was not drastically changed (Fig. 6D). Meanwhile, we did not detect precocious *br* expression in the midgut of *Axn* mutant larvae (Fig. 6C and F), but detect it the fat body. These results indicate that *Axn* mutant induces precocious *br* expression tissue-specifically.

### *Met*, *gce* and *Kr-h1*expression is suppressed in *Axn*, *slmb* and *nkd* mutants

**Figure 7:**
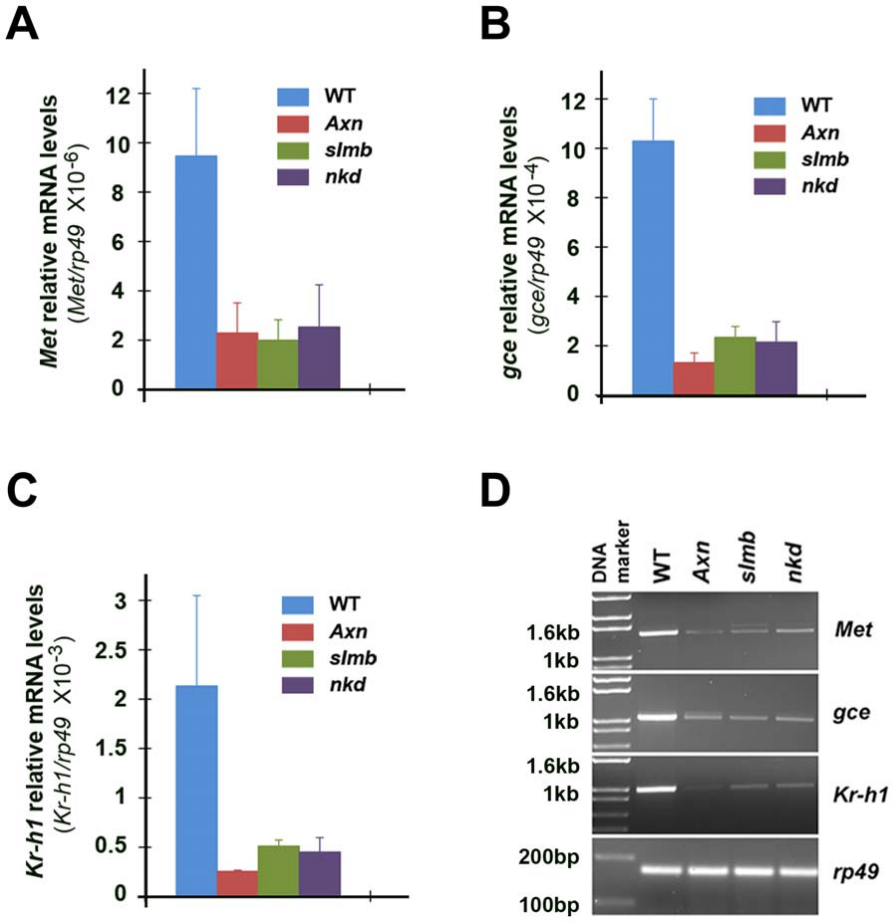
Expression of *Met*, *gce* and *Kr-h1* is reduced in the *Axn*, *slmb* and *nkd* mutants. (A) Total RNAs were extracted from *Oregon* R, *Axn^EY10228^*, *slmb^00295^*, and *nkd^2^* 2^nd^ instar larvae. The mRNA levels of *Met*, *gce* and *Kr-h1* were assessed by quantitative real-time PCR and normalized to *rp49* mRNA. Values shown are the means of 4 independent experiments ± standard deviations. (B) The same total RNAs described in [A] were used as the templates for a 30-cycle reverse transcriptional PCR. The RT-PCR products were analyzed by DNA agarose gel electrophoresis.

JH functions through Met, Gce and Kr-h1 to suppress *br* expression during the early larval stages [11], [14], [22], [23]. We next investigated whether Wnt signaling regulates *Met*, *gce* and *Kr-h1*expression. We first compared mRNA levels for *Met*, *gce* and *Kr-h1* between wild type and *Axn^EY10228^*, *slmb^00295^*, and *nkd^2^* mutants by qRT-PCR. In the *Axn*, *slmb* and *nkd* mutant 2^nd^ instar larvae, the mRNA levels of *Met*, *gce* and *Kr-h1* were only about 10–30% of that in wild type at the same stage (Fig. 7A–C). Similarly, when reverse transcriptional PCR was carried out for 30 cycles, the *Met*, *gce* and *Kr-h1* mRNA levels were also obviously reduced in the *Axn*, *slmb*, and *nkd* mutant 2^nd^ instar larvae (Fig. 7D). These results suggest that *Met*, *gce* and *Kr-h1*expression are suppressed in *Axn*, *slmb* and *nkd* mutants, which results in precocious *br* expression.

### Gain-of-function of *arm* activates *br* and suppresses *Met*, *gce* and *Kr-h1* expression

**Figure 8:**
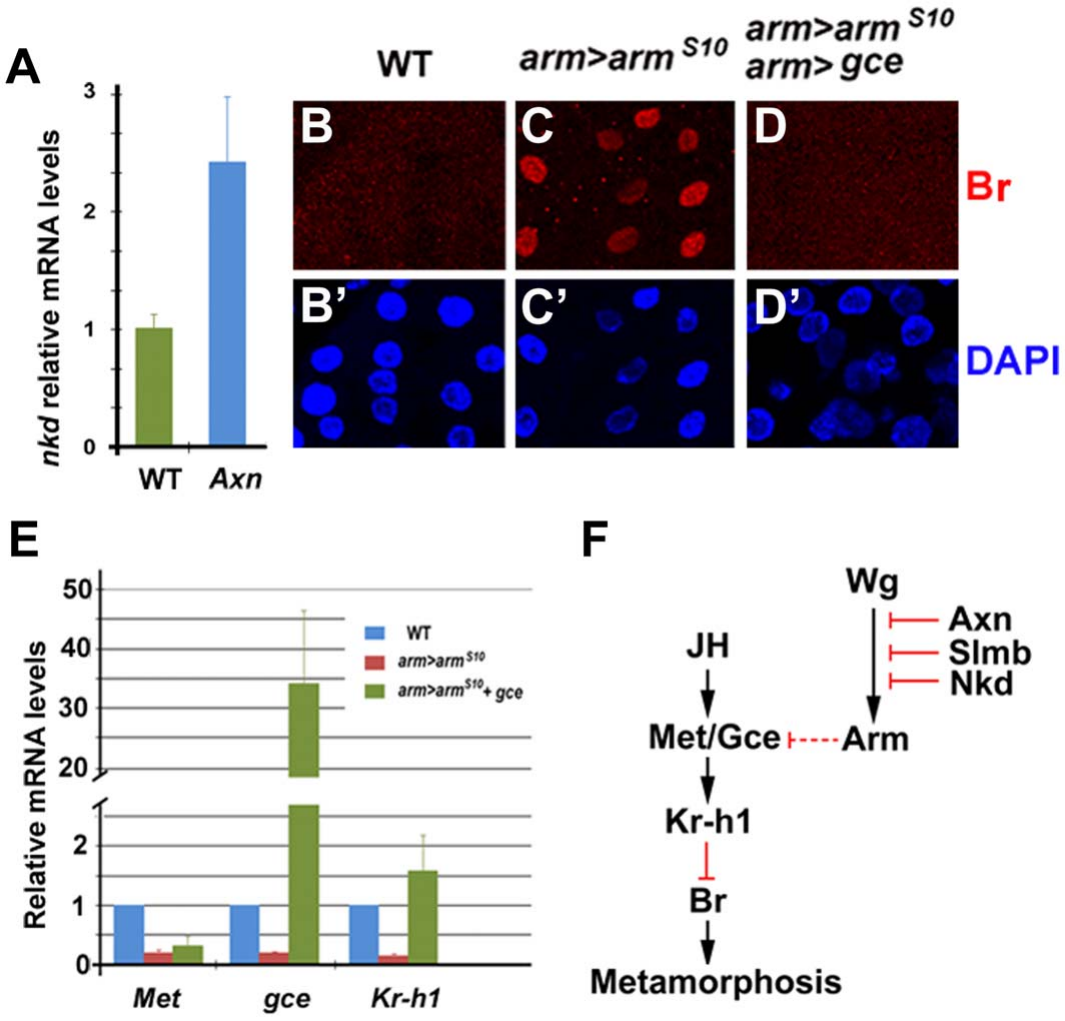
Gain-of-function *arm* mutation suppresses *Met*, *gce* and *Kr-h1*expression and induces precocious *br* expression. (A). Total RNAs were extracted from *Oregon* R and *Axn^EY10228^* 2^nd^ instar larvae. The mRNA levels of *nkd* were assessed by quantitative real-time PCR and normalized to *rp49* mRNA. Values shown are the means of 4 independent experiments ± standard deviations. (B–D). Fat bodies of 2^nd^ instar larvae were stained with a Br-core antibody (red) and DAPI (blue). (E). Total RNA was extracted from the 2^nd^ instar larvae. The mRNA levels of *Met*, *gce* and *Kr-h1* were assessed by qRT-PCR and normalized to *rp49* mRNA. Values shown are the means of 4 independent experiments ± standard deviations. Genotypes include: wild type; *arm-GAL4/UAS-arm^S10^* and *arm-GAL4/UAS-arm^S10^,UAS-gce/+*. (F). As described in the text, the proposed model illustrates the cross-talk between the Wnt and JH signaling pathways.

Because Axn, Slmb, and Nkd negatively affect Wnt signaling activity [28]–[30], increased Wnt signaling activity was expected in the *Axn*, *slmb* and *nkd* mutants. We tested the Wnt signaling activity in the Axn mutant larvae by detecting *nkd* expression. *Drosophila nkd* is an inducible antagonist for the Wnt signal. Its expression is induced by Wnt activity and its product in turn represses Wnt activity [30]. As shown in Fig. 8A, *nkd* mRNA level in the *Axn* mutant 2^nd^ instar larvae was more than 2 times that of wild type larvae at the same stage. Therefore, we suggested that the high Wnt signaling activity accounted for precocious *br* expression as well as suppression of *Met*, *gce* and *Kr-h1* transcription in the *Axn*, *slmb* and *nkd* larvae. To test this hypothesis, we examined the effects of the *arm* gain-of-function mutation on the expression of *br*, *Met*, *gce* and *Kr-h1* transcription.

Stabilization and accumulation of Arm in the cytosol increases its nucleus importation, which activates the transcription of Wnt target genes [27]. Arm^S10^ is a constitutively active form of Arm that carries a 54 amino acid deletion lacking the Shaggy phosphorylation sites and resists degradation [43]. When *UAS-arm^S10^* was driven by *arm-GAL4* to be expressed in the wild type, we detected precocious *br* expression with Br-core antibody staining in the fat bodies of 2^nd^ instar larvae (Fig. 8C). The qRT-PCR data revealed that mRNA levels of *Met*, *gce* and *Kr-h1* in the *arm-GAL4/UAS-arm^S10^* 2^nd^ instar larvae were significantly reduced to less than 20% of that in the wild type (Fig. 8E). Therefore, the phenotypes of the *arm* gain-of-function mutant are identical to that of *Axn*, *slmb* and *nkd* mutants, fully supporting that high Wnt signaling activity suppresses *Met*, *gce*, and *Kr-h1* expression and promotes *br* expression.

### Wnt signaling indirectly suppresses *Kr-h1* expression by down-regulating *Met* and *gce* expression

Our previous studies revealed that Met and Gce are functionally redundant in transducing JH signaling. The *Met-gce* double mutant can totally eliminate JH-induced *Kr-h1* expression [14]. Therefore, we investigated whether Wnt signaling indirectly suppresses *Kr-h1* expression by down-regulating *Met* and *gce*. We co-expressed *arm^S10^* and *gce* in wild type flies and examined *br*, *Met*, *gce* and *Kr-h1* expression. When *UAS-arm^S10^* and *UAS-gce* were driven by *arm-GAL4*, the precocious *br* expression induced by *arm-GAL4/UAS-arm^S10^* was totally suppressed, indicated by the absence of Br proteins in the nuclei of 2^nd^ instar larval fat body cells (Fig. 8D). In the same organisms, the *gce* mRNA level was increased by more than 30 times; the *Kr-h1* mRNA level was restored to ∼150% that of the wild type level; and the *Met* mRNA level was ∼30% that of wild type level (Fig. 8E). These results demonstrate that ectopic expression of *gce* can block Arm^S10^-mediated *Kr-h1* suppression, but does not affect Arm^S10^-mediated *Met* suppression. We conclude that Wnt signaling indirectly regulates *Kr-h1* expression by down-regulating *Met* and *gce* (Fig. 8F)

Taken together, our genetic screen and further investigations demonstrate that Wnt signaling suppresses transcription of the potential JH receptors *Met* and *gce*, which reduces JH signaling activity as evident by the reduced *Kr-h1*expression and precocious *br* expression. This study reveals that Wnt signaling cross-talks with JH signaling in mediating insect metamorphosis.

## Discussion

### JH is required to repress *br* expression during the early larval stages of *Drosophila*

The ‘status quo’ action of JH in controlling insect metamorphosis is conserved in hemimetabous and most holometabous insects. However, the larval-pupal transition in higher Diptera, such as *Drosophila*, has largely lost its dependence on JH. For instance, in most insects, the addition of JH in larvae at the last instar causes the formation of supernumerary larvae. However, exogenous JH does not prevent pupariation and pupation in *Drosophila*, and instead only disrupts the development of the adult abdominal cuticle and some internal tissues [36], [44]. The molecular mechanisms underlying these differential responses to JH are not clear.

Broad is a JH-dependent regulator that specifies pupal development and mediates the ‘status quo’ action of JH [7]. In the relatively basal holometabolous insects, such as beetles and moths, JH is both necessary and sufficient to repress *br* expression during all of the larval stages [9], [10]. Our studies revealed that JH is also required during the early larval stages in the more derived groups of the holometabolous insects, such as *Drosophila*, but it is not sufficient to repress *br* expression at the late 3^rd^ instar. During the early larval stages, overexpression of the JH-degradative enzyme JHE, reduction of JH biosynthesis or disruption of the JH signaling always causes precocious *br* expression in the fat body. However, exogenous JHA treatment can not repress *br* expression in the fat body of late 3^rd^ instar larvae (data not shown). The molecular mechanism underlying the developmental stage-specific responses of the *br* gene to JH signaling remains to be clarified.

### Interactions between Wnt and JH signaling pathways

As our knowledge of signal transduction increases, the next step is to understand how individual signaling pathways integrate into the broader signaling networks that regulate fundamental biological processes. In vertebrates, Wnt signaling has been found to interact with different hormone signaling pathways to mediate various developmental events. For example, the Wnt/beta-catenin signaling pathway interacts with thyroid hormones in the terminal differentiation of growth plate chondrocytes [45] and interacts with estrogen to regulate early gene expression in response to mechanical strain in osteoblastic cells [46], [47]. In insects, both Wnt and JH signaling are important regulatory pathways, each controlling a wide range of biological processes. Here, we report for the first time that the Wnt signaling pathway interacts with JH in regulating insect development. During the *Drosophila* early larval stages, elevated Wnt signaling activity in the *Axn*, *slmb*, *nkd* mutants and *arm-GAL4/UAS-arm^S10^* flies represses *Met* and *gce* expression, which down-regulates *Kr-h1* and causes precocious *br* expression in the fat body. Ectopic expression of *UAS-gce* in the *arm-GAL4/UAS-arm^S10^* larvae is sufficient for restoring *Kr-h1* expression and then repressing *br* expression.

Arm is a co-activator that interacts with *Drosophila* TCF homolog Pangolin (Pan), a Wnt-response element-binding protein, to stimulate expression of Wnt signaling target genes [48]. In the absence of nuclear Arm, Pan interacts with Groucho, a co-repressor, to repress transcription of Wingless-responsive genes [49]. Upon the presence of nuclear Arm, it binds to Pan, converting it into a transcriptional activator to promote the transcription of Wingless-responsive genes [48]. We propose that Wnt signaling indirectly suppresses *Met* and *gce* expression by activating an unknown transcriptional repressor.

JH signaling is well known to be a systemic factor that decides juvenile versus adult commitment. Wg is a morphogen that tissue-autonomously promotes proliferation and patterning during organogenesis. Our studies show that ectopically activating Wg signaling, either by mutations of negative regulators or by the ectopic expression of Arm, results in *br* derepression via loss of Met and Gce. How and why does the localized Wg signaling regulate the global JH signaling during insect development? Our hypothesis is that though JH signaling activity is globally controlled by JH titer in the hemolymph, distinct tissues may response to JH with different sensitivity, which could be regulated by Wnt signaling-mediated *Met* and *gce* expression. Actually, we do find that precocious *br* expression is detectible in the fat body but not midgut of the *Axn* mutant 2^nd^ instar larvae. This is one line of evidence to support that Wnt signaling regulates *Met* and *gce* expression in a tissue-specific manner.

## Materials and Methods

### Fly Strains and Genetics

All *Drosophila* strains were grown on standard cornmeal/molasses/yeast food at 25°C. *Oregon* R strain was used as wild type. The *GAL4-PG12* line was a gift from H.-M. Bourbon [50]. *UAS-gce* was a gift from T. Wilson [13]. All lethal mutant lines used in the genetic screen as well as *arm-GAL4* and *UAS-arm^S10^* were obtained from the Bloomington *Drosophila* Stock Center. To generate *hs-jhe* transgenic flies, *jhe* cDNA was isolated by RT-PCR (primer sequences: forward 5′- ATTCCGCGGCAAatgctacaactgctgcttcttg-3′ and reverse 5′- ATTTCTAGAttacttttcgttgagtatatgc-3′), and inserted into *pCaSpeR-hs*. Transgenic fly lines were generated by *P* element-mediated germline transformation at Rainbow Transgenic Flies, Inc (Camarillo, CA). Heat-shock treatment of *hs-jhe* flies was performed for 45 minutes at 37°C twice a day starting at larva hatching.

### Immunohistochemistry and Microscopy

Immunohistochemistrical analysis of larval fat bodies was performed as previously described [42]. Florescence signals were captured with a Zeiss LSM510 confocal microscope (Carl Zeiss) and processed with Adobe Photoshop.

### JHA Treatment

The JHA pyriproxyfen (Sigma) was dissolved in 95% ethanol to yield a 300 ppm stock solution. The JHA-containing fly food was prepared by adding the JHA stock solution to the standard cornmeal-molasses-yeast food at 50–55°C to a final concentration of 0.1 ppm.

### Western Blotting

Protein extracts isolated from the 2^nd^ instar larvae were analyzed by standard SDS–PAGE and Western blotting. The expression of β-tubulin was used as a loading control. Br mouse monoclonal antibody Br-core (25E9.D7) [32] and β-tubulin mouse monoclonal antibody (AA12.1) were obtained from the Developmental Studies Hybridoma Bank at the University of Iowa.

### qRT-PCR

Total RNAs were prepared from the 2^nd^ instar larvae using the RNeasy Mini Kit (Qiagen). Quantitative real-time PCR (qRT-PCR) was performed using the LightCycler 480 SYBR Green I Master Kit (Roche). The mRNA levels of different genes were normalized to *rp49* mRNA with 4 replicates for each sample. The primers used in this study are listed in Table 1.

**Table 1:** Primers used in qRT-PCR.

Genes	Purpose	Forward Primers	Reverse Primers
***Met***	qRT-PCR	5′-GCCAGAACCCTATCAGTTGG-3′	5′-AGCAGACGGTAGCAGCTCTC-3′
***gce***	qRT-PCR	5′-GATCCGAATCCGATGACTTC-3′	5′-GAATTTGCGGGAACAGAGTC-3′
***Kr-h1***	qRT-PCR	5′-CTCTGCACGTCAGCGATCTA-3′	5′-AACGTCCGGATTGGGTAGAG-3′
***rp49***	qRT-PCR	5′-GACAGTATCTGATGCCCAACA-3′	5′-CTTCTTGGAGGAGACGCCGT-3′
***Met***	RT-PCR	5′-GCAGTGATCTGGAGGAGGAG-3′	5′-ACCGTCTCTGCTGAATCCAC-3′
***gce***	RT-PCR	5′-CGTCGATCTCGAGGAGGATA-3′	5′-GATCAGCTGCTGTTTGAGCA-3′
***Kr-h1***	RT-PCR	5′-CGGAGCAGATCCCTATCAGT-3′	5′-AACGTCCGGATTGGGTAGAG-3′
***rp49***	RT-PCR	5′-GACAGTATCTGATGCCCAACA-3′	5′-CTTCTTGGAGGAGACGCCGT-3′
